# Electrochemical–LSPR Multiplexed Immunoassay with a Multi-Analyte Index for Sepsis-Associated Biomarkers

**DOI:** 10.1021/acsmeasuresciau.6c00255

**Published:** 2026-09-06

**Authors:** Zoe Bradley, Abhijit Ganguly, Pagona Papakonstantinou, Nikhil Bhalla

**Affiliations:** Nanotechnology and Integrated Bioengineering Centre (NIBEC), School of Engineering, 42259 Ulster University, 2−24 York Street, Belfast, Northern Ireland BT15 1AP, U.K.

**Keywords:** LSPR, Electrochemistry, Multianalyte-assay, Sepsis, Biosensors

## Abstract

Multiplexed immunoassays frequently face challenges associated with signal cross-reactivity, matrix effects, and the integration of analytes with disparate concentration ranges and transduction efficiencies. Here, we report an electrochemical multiplexed immunoassay implemented on a four-working-electrode screen-printed carbon electrode (4-WE SPCE) platform, which was complemented with localized surface plasmon resonance (LSPR). Distinct redox probes—methylene blue (MB), cysteine (Cys), and ruthenium hexamine chloride (RuHex) were assigned to individual working electrodes, enabling parallel differential pulse voltammetry (DPV) measurements of three inflammatory biomarkers in phosphate-buffered saline (PBS) and 50% human serum. Blank samples were analyzed separately, and the nonzero calibration ranges were 0.5–20 ng mL^–1^ for procalcitonin (PCT) and 0.5–20 μg mL^–1^ for both C-reactive protein (CRP) and serum amyloid A (SAA). The bioassay demonstrated concentration-dependent current suppression, with calculated limits of detection (LoDs) of 0.0255 ng mL^–1^ for PCT, 1.60 μg mL^–1^ for CRP, and 0.0670 μg mL^–1^ for SAA in PBS, and 6.99 ng mL^–1^ for PCT, 0.0556 μg mL^–1^ for CRP, and 0.193 μg mL^–1^ for SAA in 50% human serum. Minimal cross-reactivity between biomarkers was observed, supporting analytical selectivity, while the measurements showed repeatability across replicate experiments (*n* ≥ 3). Gold nanoparticle (AuNP) surface engineering enhanced electron-transfer kinetics and antibody immobilization, producing analyte-specific electrochemical signatures. Complementary LSPR measurements provided an orthogonal optical readout that offered evidence of antibody–antigen binding at the plasmonic interface, strengthening confidence in assay specificity and performance. To support multiplexed signal integration, a dimensionless multianalyte index (MAI) based on normalized concentrations was introduced as a proof-of-concept analytical framework for combining heterogeneous biomarker signals. This MAI framework enables standardized interpretation of multiplexed electrochemical measurements using sepsis-associated inflammatory biomarkers, highlighting its potential applicability to broader multiplexed immunoassay platforms.

## Graphical abstract

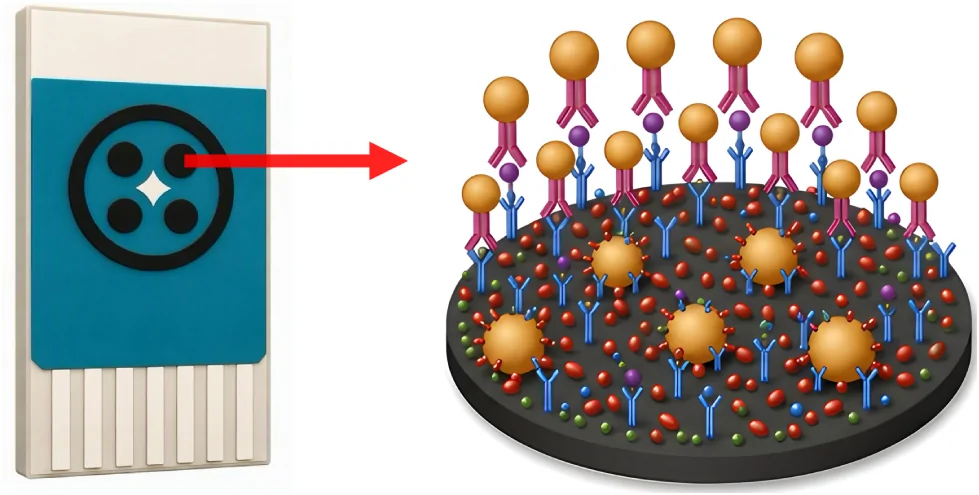

## Introduction

Sepsis remains a major cause of mortality worldwide in intensive care units (ICUs)^1,2^ and patient outcomes depend critically on the rapid recognition of the dysregulated host response that defines the condition,^3^ with survival rates for severe forms decreasing by 8% every hour.^4^ Current diagnostic methods rely heavily on blood cultures,^5^ which are limited by long turnaround times, poor sensitivity, and inadequate support for early evaluation of illness severity.^6^ Although blood circulating protein biomarkers including procalcitonin (PCT), C-reactive protein (CRP), and serum amyloid A (SAA) have emerged as informative indicators of sepsis severity and systemic inflammation, currently there is no single biomarker that is sufficient to reliably capture the heterogeneous host response associated with sepsis in terms of sensitivity and specificity.^7,8^ As a result, multiplexed approaches are increasingly sought after to improve diagnostic accuracy and aid clinical decision-making.^9^ Multiplexed electrochemical biosensing offers an alternative solution for rapid sepsis assessment^10,11^ providing high sensitivity, low cost, and portability.^12,13^ When gold nanoparticles (AuNPs) are incorporated in electrochemical bioassays, they can enhance performance by increasing the effective surface area for biorecognition^14^ and improve electron-transfer kinetics.^15^ Furthermore, plasmonic AuNPs can be used with complementary optical approaches such as localized surface plasmon resonance (LSPR) to provide a more robust diagnostic approach.^16^ From an analytical perspective, multiplexed immunoassays remain challenging due to differences in biomarker concentration scales, transduction efficiencies, and susceptibility to matrix-dependent signal distortion.

Here, we present a multiplexed electrochemical bioassay that utilizes AuNP–enabled surface engineering and redox-probe-based voltammetry to quantify PCT, CRP, and SAA. The bioassay is implemented on a 4-working electrode screen-printed carbon electrode (4-WE SPCE), with each biomarker assigned to a dedicated WE and an additional WE serving as a blank. Because the capture antibodies are electrochemically silent, a sandwich bioassay architecture was employed on the WE, in which each biomarker was paired with a distinct redox probe: cysteine (Cys) for CRP, methylene blue (MB) for PCT, and ruthenium hexamine chloride (RuHex) for SAA. Cyclic voltammetry (CV) was used for WE characterization and activation, while differential pulse voltammetry (DPV) provided the analytical readout. In this configuration, target binding reduces the amount of accessible redox species, producing a concentration-dependent decrease in the voltammetric signal. The 4-WE SPCEs were further enhanced through hierarchical micro/nanostructuring to improve electron-transfer characteristics and increase antibody loading capacity. The use of independent redox probes enabled clear biomarker-specific signatures, allowing parallel quantification on a single SPCE. LSPR measurements served as an independent optical complementary readout of antigen binding at the NP-modified interface.

We note that while individual biomarkers such as PCT, CRP, and, SAA provide valuable information on inflammatory status, their interpretation in isolation is limited by interpatient variability, overlapping concentration ranges, and differing temporal responses during disease progression.^7,17–19^ From an analytical standpoint, multiplexed immunoassays therefore require strategies that enable systematic integration of heterogeneous signals rather than reliance on absolute thresholds for individual analytes. To address this challenge, we introduce a dimensionless multianalyte index (MAI) that consolidates normalized electrochemical responses into a single composite metric. Individual biomarker signals are mapped onto normalized calibration domains and combined using adjustable weighting factors, enabling standardized data fusion while preserving relative analytical sensitivity. This framework provides a generalizable approach for interpreting multiplexed electrochemical immunoassay data and is demonstrated here using sepsis-associated inflammatory biomarkers.

The present study represents a significant advancement beyond our previously reported multiplexed microarray lateral flow device (μALFD) architecture for inflammatory biomarker detection.^20^ While the earlier μALFD system relied primarily on optical plasmonic intensity measurements, the current platform enables independent electrochemical discrimination through distinct DPV redox peaks assigned to each biomarker channel. This provides a greater degree of multiplexed analytical information compared with optical intensity-based test spot analysis alone. Specifically, each biomarker produces a distinct electrochemical signature through its assigned redox probe, whereas the LSPR response in this configuration provides complementary optical evidence of binding rather than independent analyte-specific discrimination.

Building on this framework, the presented platform delivers a unified sensing and analysis workflow for the multiplexed measurement of sepsis-associated biomarkers. The combination of multiplexed electrochemical measurements with LSPR-based validation enables robust complementary assessment of biomolecular interactions while maintaining analytical sensitivity in both phosphate-buffered saline (PBS) buffer and human serum matrices. Importantly, 4-WE SPCE architecture enables multiple biomarker-specific measurements within a compact electrode format. Furthermore, through integrating multiplex biomarker electrochemical sensing with a complementary optical readout and normalized signal processing, the platform provides a proof-of-concept analytical framework for multiplexed biomarker detection. By integrating multibiomarker sensing with complementary optical readout and data-ready signal processing, the platform establishes a scalable foundation for future development toward practical multiplexed sensing applications in sepsis management. The novelty of this study lies in the integration of multiplexed electrochemical sensing and orthogonal LSPR validation within a compact 4-WE SPCE platform. The system combines electrochemically distinct redox probes for parallel biomarker discrimination together with a generalized MAI framework for normalized signal integration across heterogeneous biomarkers.

## Results and Discussion

The surface of the carbon WE on the SPCE was functionalized to aid a sandwich bioassay in a stepwise manner, as depicted in Figure 1a. The multiplexed sandwich immunoassay was constructed by initially adding glutaraldehyde to the carbon WEs from which an aldehyde group bonded to the WE, leaving another aldehyde group available for binding to the antibody’s available amine group. Following this, AuNPs were added to enhance electrochemical performance and increase the available surface area for biomolecule immobilization. The capture antibody was then added to the glutaraldehyde-AuNP-modified electrode followed by glycine which capped the ends of unbound glutaraldehyde chains to inhibit nonspecific binding. Electrochemically distinct redox probes were assigned to individual WEs and target antigen incubation was then carried out to enable formation of the capture antibody–antigen complex. Finally, AuNP-conjugated detection antibodies were introduced to complete the sandwich bioassay architecture. The AuNP on the conjugated detection antibody provided a plasmonic label for complementary LSPR assessment of binding events. AuNP deposition was characterized by using a scanning electron microscope (SEM) in Figure 1(b–d) which respectively displays a bare carbon WE showing the characteristic heterogeneous microroughness of printed carbon ink, a carbon WE functionalized with glutaraldehyde and AuNPs and a further magnified image to show the spherical shape, monodispersity and 35 nm diameter of the AuNPs. This hierarchical micro/nanoscale surface morphology was designed to improve overall bioassay performance. Glutaraldehyde is used for covalent capture antibody immobilization on the WE surface.^21^ AuNPs in particular are known to increase the WE’s effective surface area, which aids electron-transfer efficiency, and provides a chemically favorable surface for stable, oriented antibody immobilization.^22^ As antibodies lack redox activity,^23^ indirect monitoring of antibody–antigen binding was achieved by pairing each biomarker with a specific redox probe. The electrochemical measurement setup is shown in Figure S1. As indicated in the preceding text, three redox probes MB for PCT, Cys for CRP, and RuHex for SAA were used. Electrochemical characterization of the redox probes in the absence and presence of AuNPs on the surface of the 4-WE SPCE was performed using CV followed by DPV as illustrated in Figure 2a–c and Figure 2d–f, respectively.

**Figure 1:**
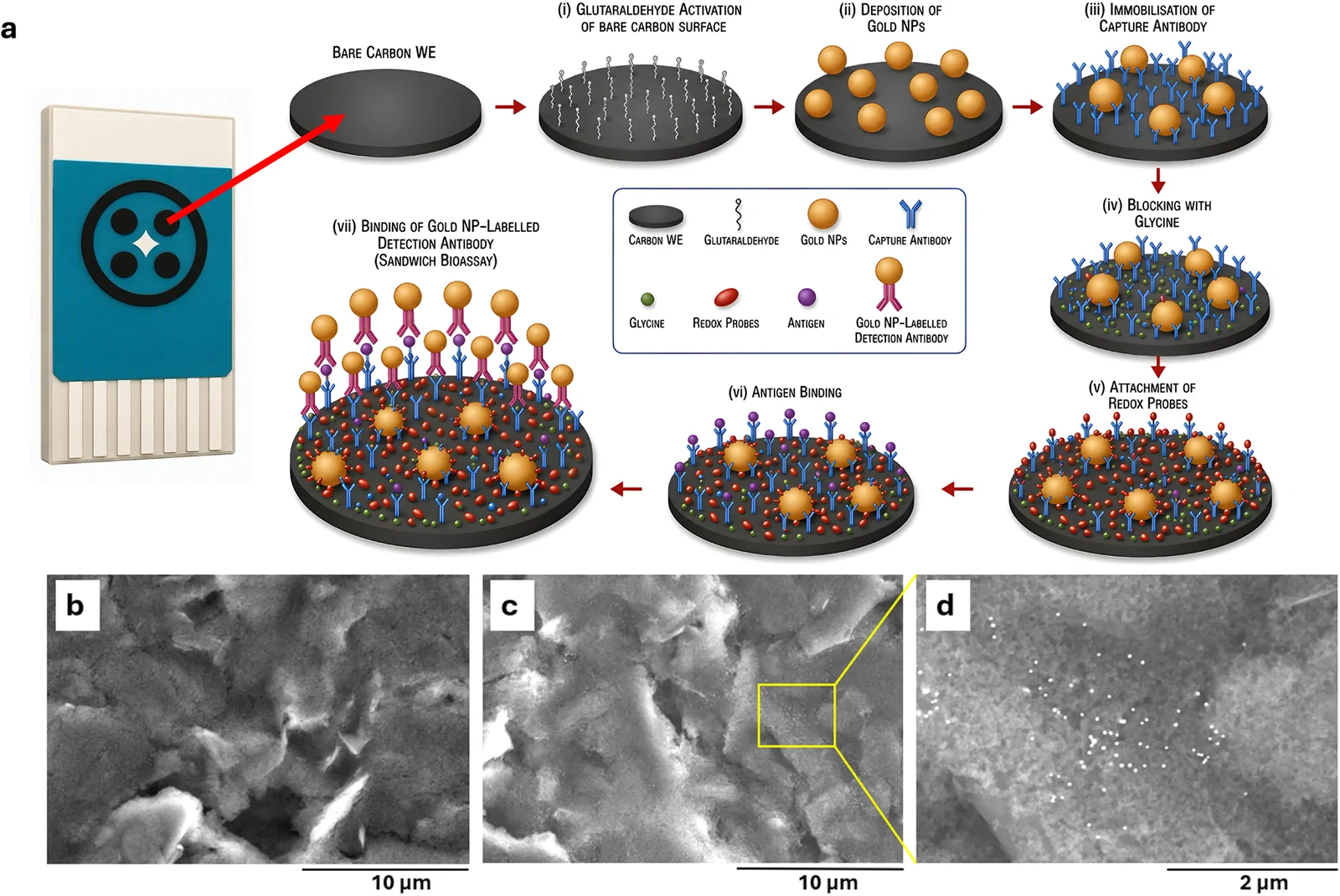
Surface functionalization of the 4-WE SPCE: a) Schematic illustrating the stepwise modification of the carbon WE: glutaraldehyde activation of the bare carbon surface used for capture antibody immobilization, deposition of AuNPs, immobilization of the capture antibody, blocking with glycine, attachment of a redox probe, antigen binding, and final binding of the AuNP–labeled detection antibody to form a sandwich bioassay, b) SEM characterization of the bare WE surface, c) WE surface functionalized with glutaraldehyde and AuNPs, and d) magnified WE surface functionalized with glutaraldehyde and AuNPs. Schematic in panel a, is made with FigureLabs tool.

**Figure 2:**
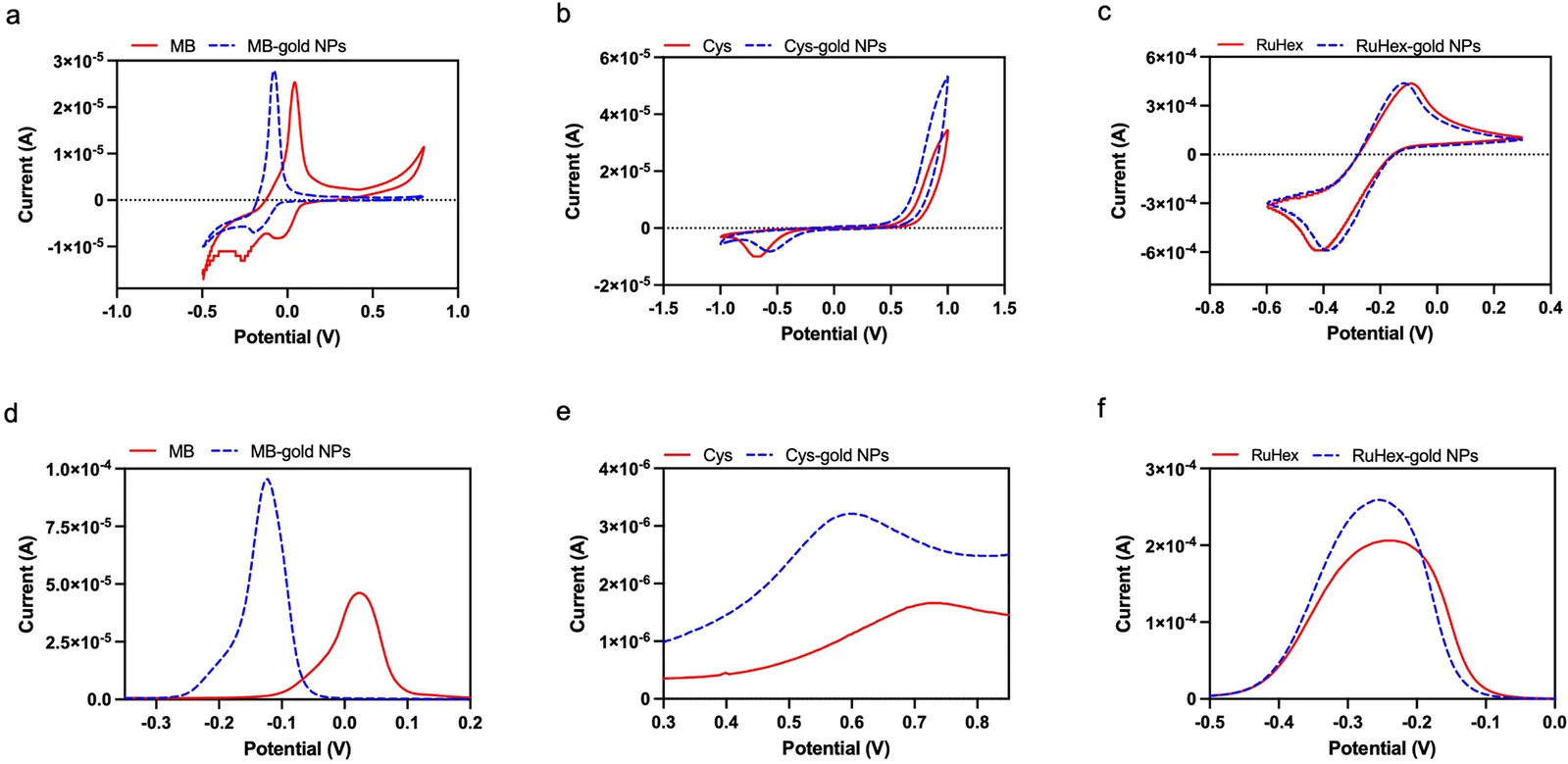
Electrochemical characterization of the redox probes on the 4-WE SPCE platform in the absence and presence of AuNPs. CV responses are shown for (a) methylene blue (MB), (b) cysteine (Cys), and (c) ruthenium hexamine chloride (RuHex), while DPV responses are shown for (d) MB, (e) Cys, and (f) RuHex. The AuNP-modified electrodes exhibit enhanced redox peak currents and altered peak potentials compared with the unmodified electrodes, indicating improved electron-transfer behavior and supporting the suitability of the AuNP-functionalized 4-WE SPCE platform for redox-probe-based multiplexed biomarker detection.

CV profiles exhibited clear and reproducible redox peaks for all probes, while incorporation of AuNPs resulted in enhanced redox peak currents accompanied by a negative shift in peak potentials, indicative of faster and more favorable electron transfer between the WE and its associated redox probes. DPV, selected for analytical measurements due to its higher sensitivity, yielded sharp and well-resolved peaks across all redox probes. The use of redox probes with distinct peak potentials enabled straightforward differentiation of individual biomarkers in a multiplexed format, with redox probe-dependent responses observed. Collectively, these results were consistent with integration of distinct redox signatures for biomarker-specific quantification in the multiplexed platform. The use of redox probes with well-separated electrochemical potentials minimized signal cross-reactivity and enabled biomarker-specific analytical responses across the individual working-electrode channels within a multiplexed assay.

DPV measurements demonstrated an inverse relationship between redox peak current and antigen concentration for all three biomarkers, consistent with the expected surface-masking mechanism of the sandwich bioassay. This inverse response arises from progressive masking of the redox probe following antibody–antigen sandwich-complex formation. Concentration-dependent suppression trends for PCT, CRP, and SAA are shown in Figure 3a–c for measurements in PBS and 50% human serum. Full DPV voltammograms are presented in Figure S2. In both media, increasing antigen concentration produced gradual decreases in current, consistent with formation of the sandwich antibody–antigen complexes. Blank samples were analyzed separately, while the nonzero log–linear calibration ranges were 0.5–20 ng mL^–1^ for PCT and 0.5–20 μg mL^–1^ for CRP and SAA.

**Figure 3:**
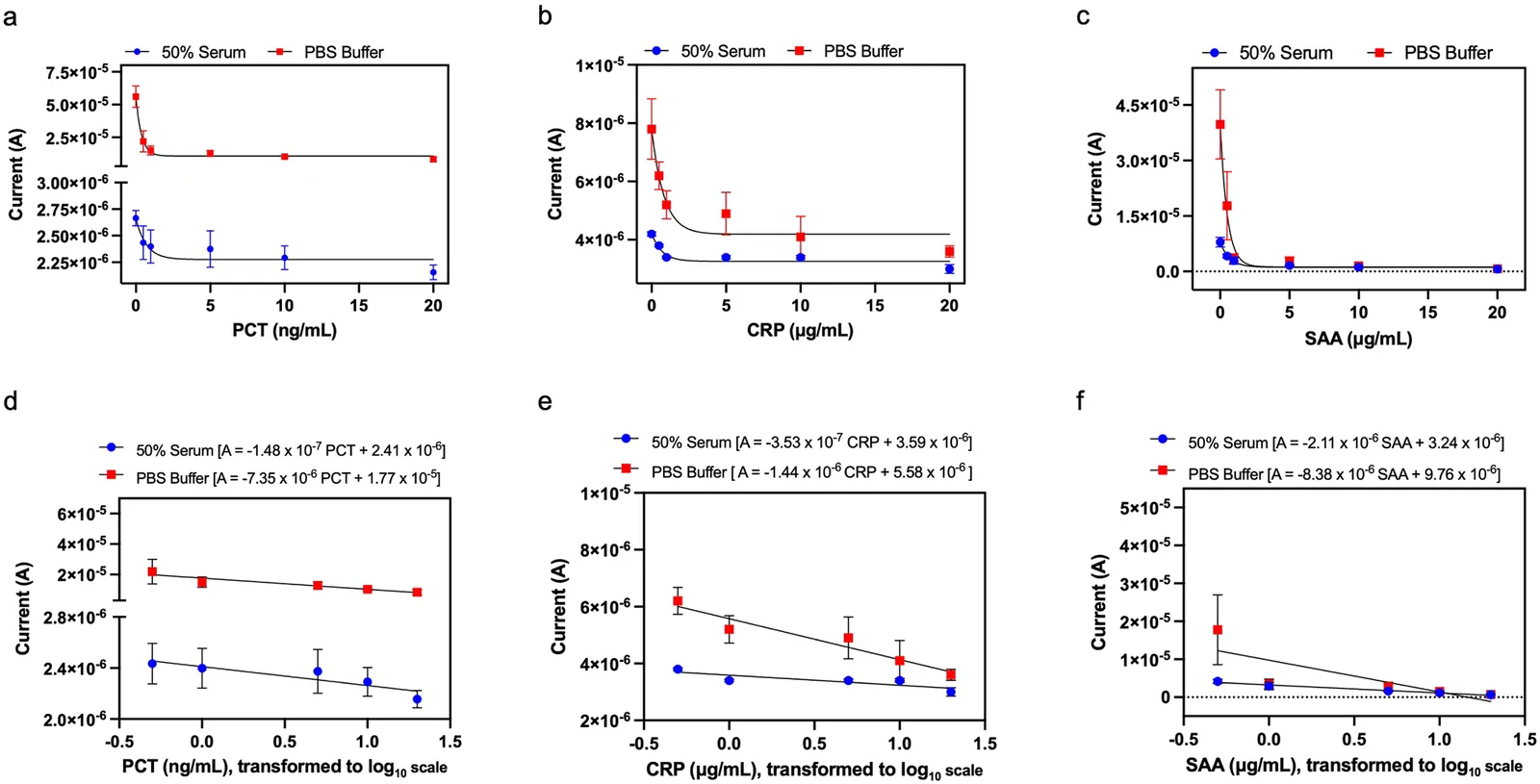
Comparison of DPV responses obtained from sandwich bioassays performed in PBS buffer and 50% human serum. (a–c) Peak current responses for PCT, CRP, and SAA bioassays, respectively, measured across increasing antigen concentrations. (d–f) Corresponding linear regression analyses comparing the peak current responses obtained in PBS buffer and 50% human serum for PCT, CRP, and SAA, respectively. The decrease in peak current with increasing antigen concentration is consistent with progressive redox-probe masking following sandwich antibody–antigen complex formation. Error bars in (a–f) represent the standard error of the mean (SeM) from replicate measurements (*n* ≥ 3).

Linear regression analysis in Figure 3d–f demonstrated matrix-dependent differences between PBS and 50% human serum. Because the signal decreased with increasing concentration, the lower-tail expressions LoB = *A̅*_blank_ – 1.645σ_blank_ and LoD_signal_ = LoB – 1.645σ_low_ were used. The signal-domain LoD was converted through the log–linear calibration equation *A* = *m*log_10_(*C*) + *b*, using log_10_(*C*_LoD_) = (*A*_LoD_ – *b*)/*m* and $C_{\mathrm{LoD}}=10^{(A_{\mathrm{LoD}}-b)/m}$ . Table 1 summarizes the resulting concentration-domain LoDs (further details in Supporting Information). The calculated LoDs in PBS were 0.0255 ng mL^–1^ for PCT, 1.60 μg mL^–1^ for CRP, and 0.0670 μg mL^–1^ for SAA. In 50% serum, the corresponding values were 6.99 ng mL^–1^, 0.0556 μg mL^–1^, and 0.193 μg mL^–1^, respectively. Values below the lowest nonzero calibration standard are reported as extrapolated analytical estimates rather than experimentally verified detection limits. Differences between PBS and serum reflect the combined effects of calibration sensitivity and matrix-dependent baseline variability. Serum proteins, salts, and other biomolecular components can influence electrochemical performance through nonspecific adsorption, partial electrode fouling, steric hindrance, and altered interfacial electron-transfer behavior.

**Table 1:** Calculated Concentration-Domain Limits of Detection for Biomarkers Tested in PBS and 50% Serum

Biomarker	PBS	50% serum
PCT	0.0255 ng mL^–1^ ^a^	6.99 ng mL^–1^
CRP	1.60 μg mL^–1^	0.0556 μg mL^–1^ ^a^
SAA	0.0670 μg mL^–1^ ^a^	0.193 μg mL^–1^ ^a^

a Calculated value below the lowest nonzero calibration standard; reported as an extrapolated analytical estimate rather than an experimentally verified detection limit.

Specificity was evaluated by testing each functionalized WE with both target and off-target antigens, seen in Figure 4a–c. Negligible signal changes were observed when unrelated antigens were introduced, whereas target antigens produced pronounced current suppression. This data suggests that the antibody pairs used for PCT, CRP, and SAA exhibit high affinity and minimal cross-reactivity, which ensures analytical independence across the different biomarkers. This specificity is particularly important for sepsis diagnostics, where biomarkers span different concentration ranges and are co-present in complex biological fluids. Although specificity and cross-reactivity studies demonstrated minimal electrochemical interference between the target biomarkers under the investigated conditions, patients with sepsis frequently exhibit elevated concentrations of interleukin-6 (IL-6),^24^ lactate,^25^ neutrophil-to-lymphocyte ratio (NLR), and white blood cells (WBC),^26^ which may contribute to nonspecific adsorption or alter interfacial electrochemical behavior in complex biological samples. While the specificity results suggest good analytical selectivity, expanded interference studies involving additional clinically relevant biomarkers will be important to further evaluate the analytical robustness of the multiplex sensing platform. Further validation experiments evaluating the contribution of the AuNP conjugate and providing supporting evidence that the observed response is associated with antibody–antigen binding rather than a dilution effect are presented in Figure S3 and Figure S4, respectively. The increased DPV response observed following incorporation of the AuNP-conjugated detection antibody (Figure S3a–c) resulted in substantially larger peak currents and well-defined concentration-dependent peaks across the tested analyte range. In contrast, measurements performed in the absence of AuNP conjugation (Figure S3b–c) exhibited only weak electrochemical responses, with no clearly distinguishable peaks observed at concentrations greater than 0.5 ng mL^-1^. This demonstrates the functional contribution of AuNP conjugation to signal enhancement within the sandwich bioassay architecture. The dilution experiment presented in Figure S4 demonstrated concentration-dependent electrochemical signal suppression consistent with progressive antibody–antigen sandwich complex formation rather than simple dilution-dependent signal variation.

**Figure 4:**
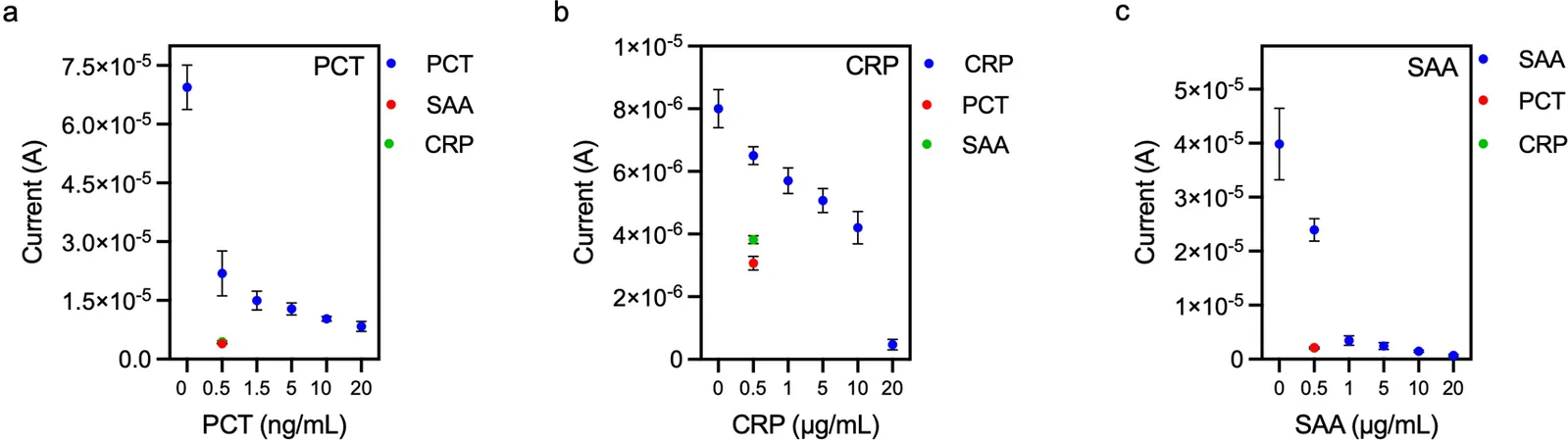
Specificity experiment: (a) PCT bioassay tested with SAA and CRP antigens, (b) CRP bioassay tested with PCT and SAA antigens, and (c) SAA bioassay tested with PCT and CRP antigens. All measurements were performed in PBS buffer. Error bars in a–c represent the SeM from multiple experimental replicates (*n* ≥ 3).

LSPR measurements provided a complementary optical response consistent with antibody–antigen binding, with increasing antigen concentrations producing an increase in absorbance for PCT, CRP, and SAA (Figure 5a–c). The increase in absorbance arose from changes in the local dielectric environment surrounding the AuNPs during antibody–antigen binding and produced strong linear correlations across the working ranges. However, because all three bioassays employed AuNPs of the same size and morphology, the LSPR peak wavelength positions were inherently identical across all three biomarkers. As a result, the LSPR response alone cannot distinguish between PCT, CRP, and SAA unless NPs with distinct sizes, shapes, or plasmonic resonance profiles are used to generate unique peak wavelengths for each target. In this work, LSPR therefore serves as a complementary, orthogonal optical validation of surface-binding events detected electrochemically rather than as a standalone multiplexed readout modality. The LSPR measurement device setup and full spectra of results for each biomarker have been presented in Figure S5 and Figure S6, respectively.

**Figure 5:**
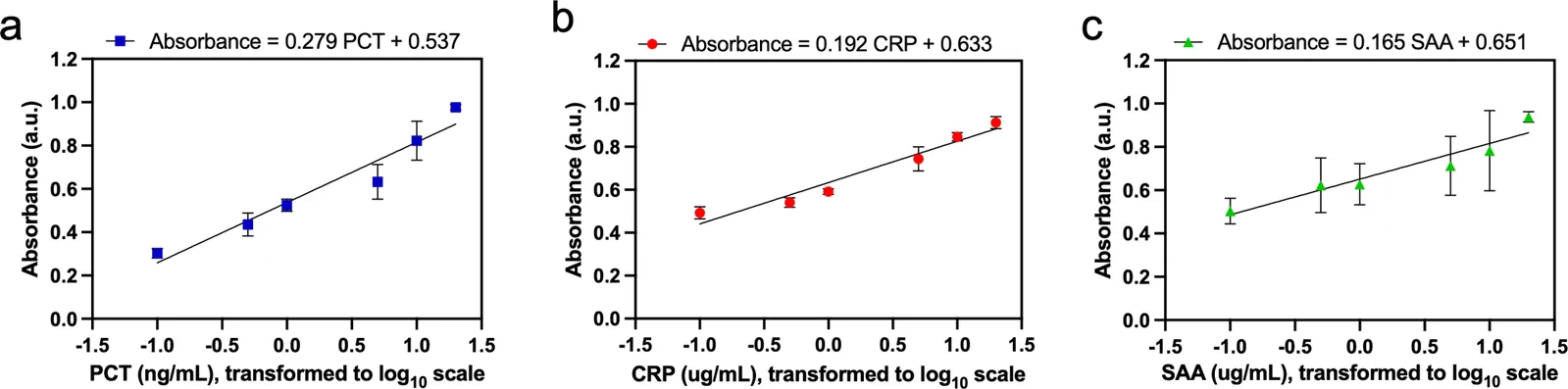
LSPR measurements completed after full sandwich assay incubation: (a) PCT, (b) CRP, and (c) SAA. All measurements were performed in PBS buffer. Error bars in a–c represent the SeM from multiple experimental replicates (*n* ≥ 3) and were fitted with linear regression analysis.

Beyond individual biomarker quantification, we introduce a MAI that consolidates responses from multiple electrochemical channels into a single dimensionless metric. Biomarker concentrations were evaluated across six defined calibration levels (X1–X6): X1 = 0, X2 = 0.5, X3 = 1, X4 = 5, X5 = 10, and X6 = 20, where PCT concentrations are expressed in ng mL^-1^ and CRP and SAA concentrations are expressed in μg mL^-1^. These calibration levels were subsequently normalized to values between 0 and 1 to enable composite signal integration. Literature-reported reference thresholds (PCT < 0.05 n, CRP < 1 μg mL^-1^, SAA < 10 μg mL^-1^) were used to define baseline healthy ranges. The MAI framework illustrates how multiplexed electrochemical signals with disparate units and dynamic ranges can be integrated into a standardized composite analytical response.

We also emphasize that the following score is an illustrative laboratory-derived construct used to demonstrate integration of the experimentally measured biomarker responses. It is not intended to represent a clinically validated diagnostic, prognostic, or disease-severity score, and the numerical values should not be interpreted as clinical decision thresholds. Using these normalized values, an illustrative composite sepsis score (*S*) is defined as a weighted sum of the three biomarkers:

1 $$S=w_{\mathrm{PCT}}\times {\mathrm{PCT}}_{n}+w_{\mathrm{CRP}}\times {\mathrm{CRP}}_{n}+w_{\mathrm{SAA}}\times {\mathrm{SAA}}_{n}$$

where *w*_PCT_, *w*_CRP_, and *w*_SAA_ denote the weight assigned to each biomarker, and the sum of all weights satisfies:

2 $$w_{\mathrm{PCT}}+w_{\mathrm{CRP}}+w_{\mathrm{SAA}}\leq 1$$

The remaining fraction can be reserved for incorporation of additional biomarkers in future iterations of the model. In this study, equal weighting was assigned to the three biomarkers:

3 $$w_{\mathrm{PCT}}=w_{\mathrm{CRP}}=w_{\mathrm{SAA}}=0.30$$

with the remaining 0.10 allocated for expansion of the panel. The equal weighting factors used here were selected as a transparent proof-of-concept approach and were not derived from clinical outcome data. Because the individual biomarker responses were first normalized to a common dimensionless scale, equal weighting provides a neutral starting point in which no biomarker is preferentially prioritized because of differences in units, concentration range, or analytical response. The resulting MAI should therefore be interpreted as a flexible analytical signal-integration framework rather than as a clinically validated sepsis-risk score.

The weighting factors may be adjusted according to the intended clinical application, biomarker performance, or clinician-defined priorities. For example, a greater weight may be assigned to PCT in applications focused on bacterial infection,^27^ whereas alternative weighting schemes may be appropriate for assessing systemic inflammation or disease severity. Determination of clinically optimized weights will require patient-level diagnostic and outcome data together with independent clinical validation, which is beyond the scope of the present proof-of-concept study.

Healthy individuals typically exhibit biomarker levels below the lowest tested concentrations of PCT < 0.05 ng mL^-1^ (X2), CRP < 1 μg mL^-1^ (X3), and SAA < 10 μg mL^-1^ (X5). Thus, a representative healthy sepsis score is

4 $$S_{\mathrm{healthy}}\approx 0.30\times 0.0025+0.30\times 0.05+0.30\times 0.5=0.17$$

In contrast, a patient presenting with biomarker levels near the severe range (X6) yields

5 $$S_{\mathrm{sepsis}}=0.30\times 1+0.30\times 1+0.30\times 1=0.90$$

These examples are mathematical illustrations based on experimentally investigated concentration levels and literature-reported reference values. They do not represent scores calculated from individual patient samples. Accordingly, the terms “healthy” and “sepsis” are used here only to illustrate relative positions within the normalized analytical range and should not be interpreted as validated clinical classifications. Indicating a high composite multianalyte response level, as visualized in Figure 6. This framework provides a scalable foundation for future clinical translation, enabling the incorporation of additional inflammatory markers as their diagnostic utility becomes established.

**Figure 6:**
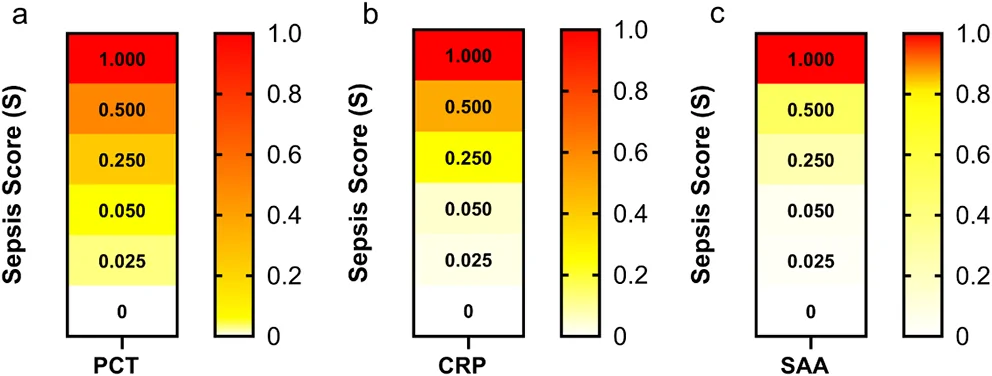
Heat maps showing the normalized concentration values of (a) PCT, (b) CRP, and (c) SAA across the defined concentration levels (X1–X6) which represents range in Sepsis Score (S). Color intensity represents increasing normalized biomarker response within the experimentally investigated range. The visualization is an illustrative analytical representation and should not be interpreted as a clinically validated measure of sepsis severity or patient risk.

The present study represents an analytical proof-of-concept and does not constitute comprehensive validation of the platform for clinical or point-of-care use. Interference testing was limited to the investigated biomarker combinations, while a broader range of clinically relevant endogenous and exogenous interferents was not evaluated. In addition, electrode-to-electrode and batch-to-batch variability, long-term storage and operational stability, and device robustness were not systematically investigated. The experiments were conducted using PBS buffer and 50% commercially obtained human serum rather than clinical patient specimens. Accordingly, further analytical validation, including expanded interference testing, independent-electrode variability studies, stability and robustness assessment, and evaluation using appropriately characterized clinical samples, will be required before clinical or point-of-care performance can be established.

## Conclusion

In summary, we developed an electrochemical multiplexed immunoassay that was complemented with optical validation. Hierarchical micro/nanostructuring with AuNPs enhanced electron-transfer kinetics and increased antibody loading, enabling detection across investigated concentration ranges in both PBS and human serum. By pairing each biomarker with a distinct redox probe, the system generated non-overlapping voltammetric signatures that supported multiplexed analysis with minimal cross-reactivity under the investigated conditions. Complementary LSPR measurements provided evidence consistent with antigen binding at the functionalized interface. Beyond individual biomarker measurement, we introduced an MAI that integrates the normalized concentrations of PCT, CRP, and SAA into a single weighted score, illustrating how multiplexed electrochemical data may be consolidated into a composite analytical metric that could, following appropriate patient-level clinical validation, potentially contribute to future risk-stratification approaches. While demonstrated here using sepsis-associated inflammatory biomarkers, the analytical strategy is applicable to multiplexed immunoassays requiring measurement, complementary validation, and data fusion across diverse biomarker panels. Further studies using clinical samples, independent-electrode and batch-level reproducibility testing, and long-term stability measurements will be required to establish analytical robustness and assess potential future clinical translation.

## Experimental Section

### Chemicals and Materials

Chemicals used included gold­(III) chloride hydrate (HAuCl_4_), sodium citrate tribasic (Na_3_Cit), hydrochloric acid (HCl), nitric acid (HNO_3_), potassium chloride (KCl), 2-(N-morpholino)­ethanesulfonic acid (C_6_H_13_NO_4_S; MES), N-(3-dimethylaminopropyl)-N′-ethylcarbodiimide hydrochloride (C_8_H_17_N_3_·HCl; EDC), N-hydroxysuccinimide (C_4_H_5_NO_3_; NHS), methylene blue hydrate (C_16_H_18_ClN_3_S·H_2_O; MB), l-cysteine (HSCH_2_CH­(NH_2_)­CO_2_H; Cys), hexaammineruthenium­(III) chloride ([Ru­(NH_3_)_6_]­Cl_3_; RuHex), hydroxylamine, glycine, glutaraldehyde solution, tris-buffered saline (TBS), Tween 20, casein, and PBS. These materials were purchased from Merck Life Science, UK. Heterobifunctional thiol–polyethylene glycol–carboxyl (HS-PEG-COOH; 5 kDa) was purchased from JenKem Technology, China. The monoclonal antibodies comprised PCT capture antibody (27A3^3^), PCT detection antibody (16B5), SAA capture antibody (SAA19^3^), SAA detection antibody (A496), CRP capture antibody (C6), and CRP detection antibody (C5). These antibodies and the recombinant PCT and SAA antigens were purchased from HyTest Ltd., Finland. CRP antigen (30C–CP1000U) was purchased from Fitzgerald, USA, and human serum (HMN803130) was purchased from BioIVT, UK. Deionized water (dH_2_O, 18.2 MΩ·cm) produced using a Milli-Q purification system was used throughout the experiments. All chemicals were used without further purification unless otherwise specified. Glassware was cleaned with aqua regia (HCl/HNO, 3:1, v/v), thoroughly rinsed withdH_2_O, and dried before use.

### AuNP Synthesis and Surface Functionalization

AuNPs with a diameter of 35 nm were prepared using the traditional Turkevich method.^28^ Briefly, 50 mL of 1 mM HAuCl_4_ solution was heated to 100 °C while being continuously stirred. Subsequently, 3 mL of 38 mM NaCit was added, resulting in a color transition from yellow to black and then to dark red. The resulting 35 nm AuNP solution was allowed to cool to room temperature under constant stirring. The AuNPs were washed twice by centrifugation at 3600 g for 30 min, the supernatant was discarded, and the pellet was resuspended in dH_2_O. AuNPs were then functionalized with a carboxyl group upon incubation with 5 mM HS-PEG-COOH in dark conditions and under continuous rotation for 2 h. The resulting carboxylated AuNPs were washed twice by centrifugation at 10600 g for 30 min. After each centrifugation, the supernatant was discarded, and the pellet was resuspended in dH_2_O.

### Covalent Conjugation

Functionalized AuNPs were conjugated to PCT, CRP, and SAA detection antibodies by combining 500 μL of carboxylated AuNPs with 200 μL of 0.1 mg mL^-1^ detection antibody in 10 μL MES, 100 μL of 100 mM MES, 200 μL of 1 mM EDC, and 400 μL of 1 mM NHS. The mixture was stirred continuously for 20 min. Next, 10 μL of hydroxylamine was added to quench the reaction, followed by an additional 10 min incubation. Subsequently, 10 mL of TBS containing 0.05% Tween 20 was added, and the solution was centrifuged at 10600 g for 20 min. The supernatant was discarded, and the final conjugates were resuspended in TBS containing 0.5% Tween 20 and 1% casein.

### Sandwich Bioassay Architecture

The WE hierarchical micro/nanoscale surface morphology was prepared by initially applying 4 μL of 2.5 mM glutaraldehyde and incubating for 1 h at 4 °C, followed by the addition of 4 μL of AuNPs, which were incubated for 1 h at 4 °C. Next, 4 μL of 1 mg mL^-1^ capture antibody was deposited on the WE and incubated for 15 min at 37 °C. Following this, 4 μL of blocking buffer (75 μM glycine in 0.1 M PBS buffer, pH 7.2) was added and incubated for 1 h at 4 °C. Then, 4 μL of redox probe solution prepared in 0.01 M KCl was applied and incubated overnight at 4 °C. The redox probes used were 5 mM MB for PCT detection, 50 mM Cys for CRP detection, and 50 mM RuHex for SAA detection. A PBS buffer wash was performed after each previous step to remove unbound materials. Subsequently, 4 μL of antigen solution, diluted in either 0.1 M PBS buffer or 50% human serum (in 0.1 M PBS buffer), starting from the lowest concentration, was applied to the WE and incubated for 15 min at 37 °C. Concentrations used were: PCT: 0, 0.5, 1, 5, 10, 20 ng mL^-1^; SAA: 0, 0.5, 1, 5, 10, 20 μg mL^-1^; and CRP: 0, 0.5, 1, 5, 10, 20 μg mL^-1^. Finally, 4 μL of 0.1 mg mL^-1^ conjugated detection antibody was added and incubated for 15 min at 37 °C before performing electrochemical measurements. Steps involving detection antibody incubation and washing were repeated sequentially for increasing antigen concentrations. For each biomarker-redox probe calibration experiment, measurements were obtained through sequential incubation of the same functionalized electrode with progressively increasing antigen concentrations, with intermediate washing steps performed between each exposure. This approach enabled monitoring of progressive redox signal suppression associated with cumulative antibody–antigen sandwich complex formation on the electrode surface. Each experiment was performed three times to evaluate reproducibility and data is presented as mean values ± SeM.

### Materials Characterization

UV–Vis absorption spectra of the AuNPs and their conjugates were recorded using a LAMBDA 365 spectrometer (PerkinElmer, UK). Measurements were taken over a wavelength range of 190–1100 nm at a scan speed of 120 nm/min, using disposable cuvettes with a 1 cm optical path length at room temperature. The NP size, morphology, monodispersity, and the 4-WE SPCE surface in its bare state and modified with glutaraldehyde with AuNPs were characterized using a Hitachi SU5000 SEM. AuNP diameters were subsequently determined using ImageJ software.

### Equipment

Electrochemical measurements were obtained using an Autolab PGSTAT204 potentiostat controlled via NOVA software (version 2.1.5). A compatible connector (CONNECT4W) and 4-WE SPCEs (4W110) were used, each comprising a carbon WE, a carbon counter electrode (CE), and a silver pseudoreference electrode (RE), all sourced from Metrohm, Switzerland. CV measurements were recorded at a scan rate of 100 mV/s for three consecutive cycles. DPV measurements were performed using a step potential of 5 mV with a step time of 0.5 s and scan rate of 10 mV/s.

### Localized Surface Plasmon Resonance Measurements

The LSPR response was measured using a custom-built optical setup designed for sample illumination and light collection. The system employed two fiber optic cables, one connected to a DH-mini-UV–VIS–NIR light source and the other to a spectrometer, both supplied by Ocean Insight (The Netherlands). An RTL-T stage from Ocean Insight enabled precise alignment of the fiber optics. Prior to signal acquisition, the spectrometer was calibrated in both dark and light conditions from which a baseline signal was obtained. For each sample, an average of three LSPR signals were recorded in absorption mode using OceanView software, allowing the wavelength-dependent light absorption by the AuNPs to be analyzed.

## Supplementary Material



## Notes

Electrochemical measurement device setup; DPV responses for multiplexed PCT, CRP, and SAA bioassays; AuNP conjugation control experiment; limit of blank and limit of detection calculations; conversion of signal-domain LoDs to concentration-domain LoDs; dilution effect studies; LSPR device setup and measurements; analytical comparison of multiplexed biosensing platforms (PDF)

## References

[1] La
Via L., Sangiorgio G., Stefani S., Marino A., Nunnari G., Cocuzza S., La Mantia I., Cacopardo B., Stracquadanio S., Spampinato S. (2024). The Global Burden of
Sepsis and Septic Shock. Epidemiologia.

[2] Rusmawatiningtyas D., Rahmawati A., Makrufardi F., Mardhiah N., Murni I. K., Uiterwaal C. S. P. M., Savitri A. I., Kumara I. F., Nurnaningsih N. (2021). Factors Associated
with Mortality of Pediatric Sepsis Patients at the Pediatric Intensive
Care Unit in a Low-Resource Setting. BMC Pediatrics.

[3] Giamarellos-Bourboulis E. J., Aschenbrenner A. C., Bauer M., Bock C., Calandra T., Gat-Viks I., Kyriazopoulou E., Lupse M., Monneret G., Pickkers P. (2024). The pathophysiology of sepsis and precision-medicine-based
immunotherapy. Nat. Immunol..

[4] Papafilippou L., Claxton A., Dark P., Kostarelos K., Hadjidemetriou M. (2021). Nanotools for sepsis diagnosis and
treatment. Adv. Healthcare Mater..

[5] Iyengar N., Dietvorst J., Ferrer-Vilanova A., Guirado G., Munoz-Berbel X., Russom A. (2021). Toward Rapid Detection of Viable Bacteria in Whole
Blood for Early Sepsis Diagnostics and Susceptibility Testing. ACS Sens..

[6] Duncan C. F., Youngstein T., Kirrane M. D., Lonsdale D. O. (2021). Diagnostic
challenges
in sepsis. Curr. Infect. Dis. Rep..

[7] Li M., Qin Y.-J., Zhang X.-L., Zhang C.-H., Ci R.-J., Chen W., Hu D.-Z., Dong S.-M. (2024). A biomarker panel
of C-reactive protein, procalcitonin and serum amyloid A is a predictor
of sepsis in severe trauma patients. Sci. Rep..

[8] Celik I. H., Hanna M., Canpolat F. E., Pammi M. (2022). Diagnosis
of Neonatal
Sepsis: The Past, Present and Future. Pediatr.
Res..

[9] Ashley B. K., Hassan U. (2021). Point-of-critical-care
diagnostics for sepsis enabled
by multiplexed micro and nano sensing technologies. Wiley Interdiscip. Rev.: nanomed. Nanobiotechnol..

[10] Lu T.-C., Yang Y.-J., Zhong Y., Qiu Q.-Z., Chen Z.-H., Chen Y.-Z., Lei Y., Liu A.-L. (2024). Simultaneous Detection
of C-Reactive Protein and Lipopolysaccharide Based on a Dual-Channel
Electrochemical Biosensor for Rapid Gram-Typing of Bacterial Sepsis. Biosens. Bioelectron..

[11] Tanak A. S., Muthukumar S., Krishnan S., Schully K. L., Clark D. V., Prasad S. (2021). Multiplexed Cytokine Detection Using an Electrochemical
Point-of-Care Sensing Device Toward Rapid Sepsis Endotyping. Biosens. Bioelectron..

[12] Bonini A., Carota A. G., Poma N., Vivaldi F. M., Biagini D., Bottai D., Lenzi A., Tavanti A., Di Francesco F., Lomonaco T. (2022). Emerging biosensing technologies towards early sepsis
diagnosis and management. Biosensors.

[13] Kaur M., Singh N. (2025). Electrochemical Biosensors
for Sepsis Detection in Liver Cirrhosis:
Advances and Insights. ACS Appl. Bio Mater..

[14] Karnwal A., Kumar Sachan R. S., Devgon I., Devgon J., Pant G., Panchpuri M., Ahmad A., Alshammari M. B., Hossain K., Kumar G. (2024). Gold nanoparticles
in nanobiotechnology:
from synthesis to biosensing applications. ACS
Omega.

[15] Maji S. K., Khan S., Mondal R. (2024). Plasmon-Accelerated Electrocatalysis
Based on Gold Nanostructures for Electrochemical Reactions and Biosensing
Applications: A Review. ACS Appl. Nano Mater..

[16] Ramasamy M., Ha J. W. (2023). Single-particle
spectroelectrochemistry: electrochemical approaches
for tuning chemical interfaces and plasmon damping in single gold
nanorods. J. Phys. Chem. Lett..

[17] Singer M., Deutschman C. S., Seymour C. W., Shankar-Hari M., Annane D., Bauer M., Bellomo R., Bernard G. R., Chiche J.-D., Coopersmith C. M. (2016). The third international
consensus definitions for sepsis and septic shock (Sepsis-3). Jama.

[18] Pierrakos C., Vincent J.-L. (2010). Sepsis biomarkers: a review. Crit. Care.

[19] Kibe S., Adams K., Barlow G. (2011). Diagnostic and prognostic biomarkers
of sepsis in critical care. J. Antimicrob. Chemother..

[20] Bradley Z., McNamee S. E., Fishlock S. J., Bhalla N., Moore T., McLaughlin J. (2025). Enhancing
Lateral Flow Device Design for Multiplexed
Plasmonic Biosensing of Inflammatory Biomarkers. IEEE Sens. Lett..

[21] Ionescu R. E. (2022). Use of
cysteamine and glutaraldehyde chemicals for robust functionalization
of substrates with protein biomarkers–an overview on the construction
of biosensors with different transductions. Biosensors.

[22] Lin Y.-H., Chen S.-H., Chuang Y.-C., Lu Y.-C., Shen T. Y., Chang C. A., Lin C.-S. (2008). Disposable
Amperometric Immunosensing
Strips Fabricated by Au Nanoparticles-Modified Screen-Printed Carbon
Electrodes for the Detection of Foodborne Pathogen *Escherichia coli* O157: H7. Biosens. Bioelectron..

[23] Prajapati, V. ; Shrivastav, P. Bioreceptors for Antigen–Antibody Interactions. Biosensors Nanotechnology; Inamuddin, Altalhi, T. , 2023; pp 371–394 10.1002/9781394167135.ch16.

[24] Carcò D., Iachelli V., Markovic U., Chisari G., Galbo F., Sciacca D., Giaimi L., Castorina P., Martorana E. (2025). Sepsis detection in hematologic and
solid tumor malignancies
using quantitative inflammatory biomarker differences in a prospective
single center study. Sci. Rep..

[25] Wang Y., Wei A., Su Z., Shi Y., Li X., He L. (2025). Characterization
of lactylation-based phenotypes and molecular biomarkers in sepsis-associated
acute respiratory distress syndrome. Sci. Rep..

[26] Charoensappakit A., Sae‑Khow K., Vutthikraivit N., Maneesow P., Sriprasart T., Pachinburavan M., Leelahavanichkul A. (2025). Immune suppressive activities of
low-density neutrophils in sepsis and potential use as a novel biomarker
of sepsis-induced immune suppression. Sci. Rep..

[27] Mahmood A., Mufti M. (2024). Diagnostic accuracy of serum procalcitonin
levels in early detection
of bacterial infections. Clin. Med..

[28] Turkevich J., Stevenson P. C., Hillier J. (1951). A study of the nucleation and growth
processes in the synthesis of colloidal gold. Discuss. Faraday Soc..

