# Supplementary material for: Electrochemical–LSPR Multiplexed Immunoassay with a Multi-Analyte Index for Sepsis-Associated Biomarkers
Source: ACS Meas Sci Au. 2026 Sep 6;6(5):1907–15. doi: 10.1021/acsmeasuresciau.6c00255 (PMC13693253; doi:10.1021/acsmeasuresciau.6c00255)
Supplement: Supplementary file 1 [file tg6c00255_si_001.pdf]

Supporting Information

Electrochemical–LSPR Multiplexed Immunoassay with a Multi-Analyte Index for Sepsis-Associated Biomarkers

Zoe Bradley[1], Abhijit Ganguly[1], Pagona Papakonstantinou[1], and Nikhil Bhalla*[1]

[1]Nanotechnology and Integrated Bioengineering Centre (NIBEC), School of Engineering, Ulster University, 2-24 York Street, Belfast BT15 1AP, Northern Ireland, United Kingdom

*Corresponding author: n.bhalla@ulster.ac.uk

Contents

1 Electrochemical measurement device setup **3**

2 DPV measurements **3**

3 Determination of the Limit of Blank and Limit of Detection **5**

4 Dilution effect **6**

5 Localised surface plasmon resonance measurements and device setup **7**

6 Analytical comparison of the present multiplexed electrochemical–LSPR immunoassay **9**

1 Electrochemical measurement device setup

Figure S1 shows the complete configuration used for all electrochemical measurements, including the Autolab potentiostat, Nova 2.1.5 software, and the connector compatible with the 4 working electrode screen printed carbon electrode (4-WE SPCE). The setup enables stable and reproducible electrical contact across the multiplex-compatible electrode system, which is essential for performing simultaneous measurements of multiple biomarkers. Future studies will include comprehensive assessment of electrode-to-electrode variability across multiple sensor batches to further establish the reproducibility and robustness of the 4-WE SPCE system.

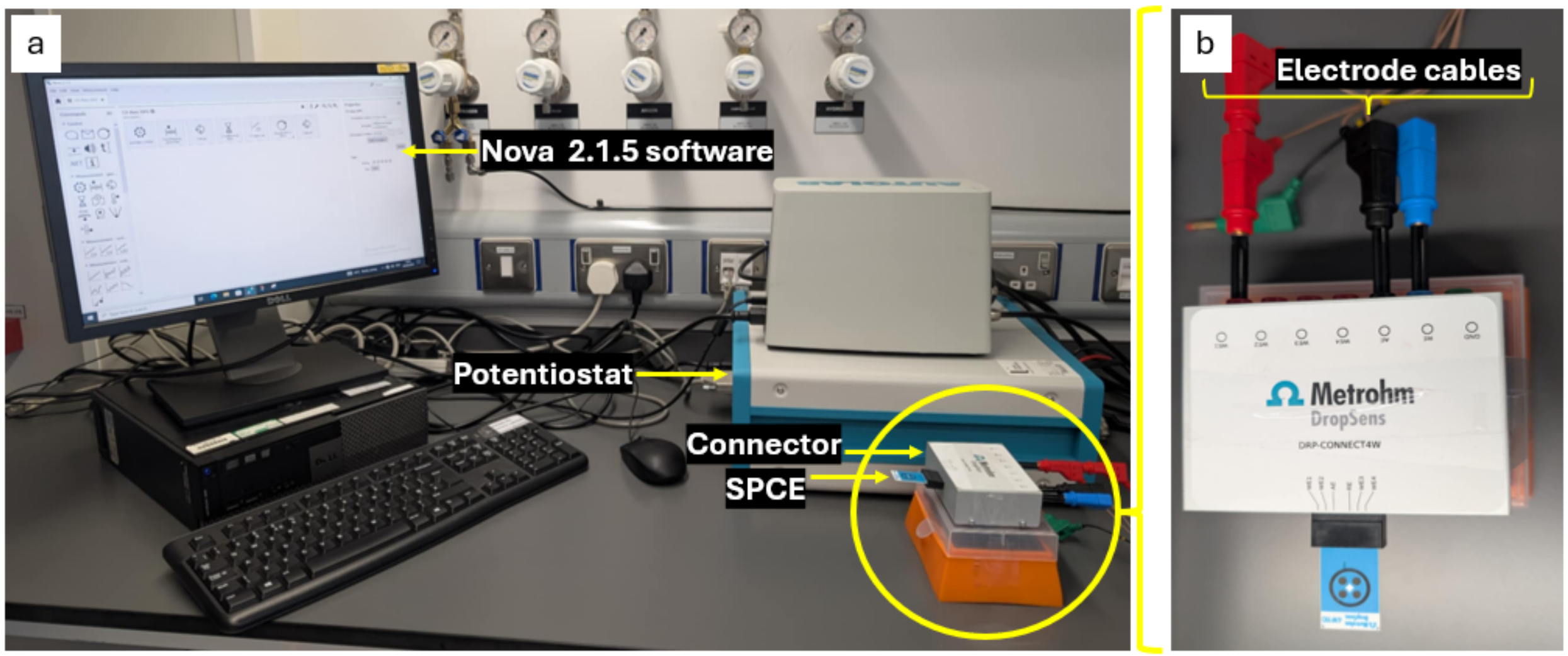

Figure S1: Electrochemical measurement device setup: a) full equipment setup including the Nova 2.1.5 software, autolab potentiostat, connector compatible with the 4-WE SPCE and SPCE, and b) birds-eye view of the electrode cables, connector compatible with the 4-WE SPCE.

2 DPV measurements

Figure S2 presents differential pulse voltammetry (DPV) responses obtained for procalcitonin (PCT), C-reactive protein (CRP), and serum amyloid A (SAA) bioassays across three redox probes, methylene blue (MB), cysteine (Cys), and ruthenium hexamine chloride (RuHex) tested in both PBS buffer and 50% serum matrices. The clear changes in peak currents between full bioassays and blanks demonstrate effective electrochemical detection of each biomarker. Importantly, the comparison between PBS buffer and diluted serum confirms that the bioassay remains functional and maintains signal integrity even in complex matrices.

Figure S3 provides a control comparison to evaluate the contribution of the gold nanoparticle (AuNP)-conjugated detection antibody to the electrochemical response of the PCT sandwich immunoassay. The complete bioassay containing the AuNP-labelled detection antibody produced a substantially higher DPV response compared with the corresponding

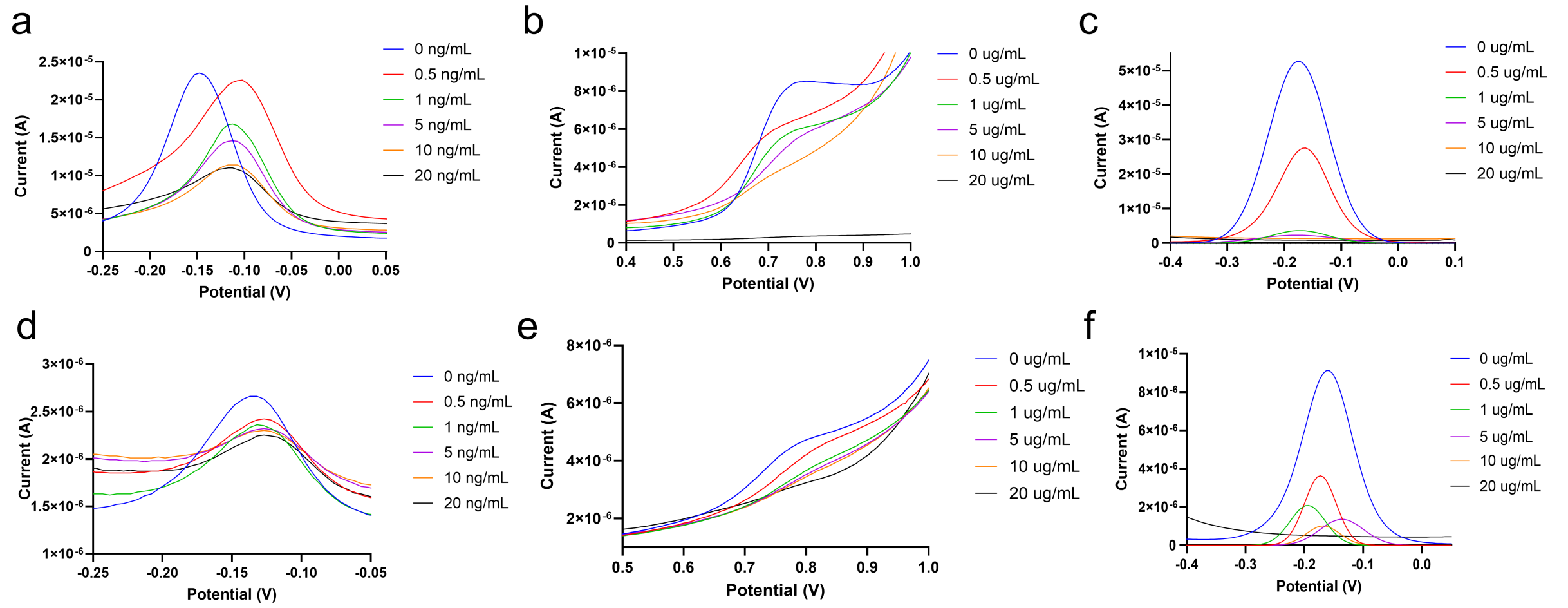

Figure S2: DPV measurements of PCT, CRP, and SAA full bioassays: (a–c) bioassays in PBS buffer with MB, Cys, and RuHex redox probes, respectively and (d–f) bioassays in 50% serum with the same corresponding redox probes.

control assay performed without AuNP conjugation. This difference demonstrates that the nanoparticle-labelled detection antibody plays an important role in enhancing the measurable electrochemical signal.

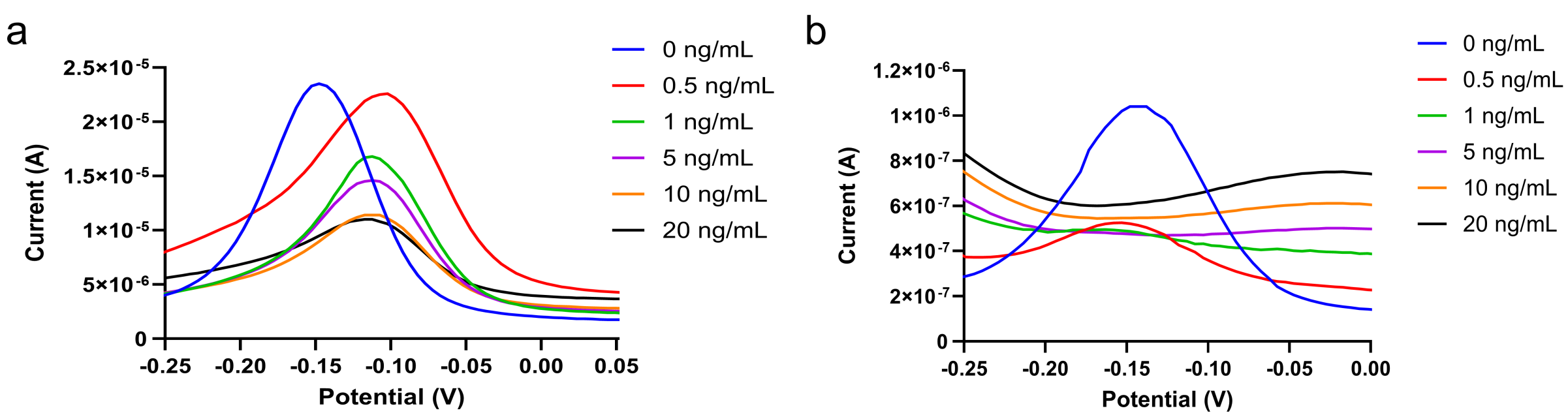

c

PCT (ng/mL)	With AuNPs (μA)	Without AuNPs (μA)
**0**	23.5	1.06
**0.5**	22.6	0.52
**1**	16.8	n/a
**5**	14.6	n/a
**10**	11.4	n/a
**20**	10.9	n/a

Figure S3: DPV response of the PCT bioassay using methylene blue (MB) as the redox probe: (a) complete sandwich bioassay in PBS buffer, reproduced from Figure S2a for direct comparison, (b) control bioassay performed without gold nanoparticle (AuNP) conjugation on the detection antibody, and (c) table comparing peak currents of parts a-b.

The reduction in signal intensity observed in the absence of AuNP conjugation confirms

that the antibody–antigen interaction alone is insufficient to generate the same magnitude of response, highlighting the contribution of the nanoparticle-assisted amplification strategy. The comparison also supports that the observed signal changes originate from formation of the complete sandwich immunocomplex rather than non-specific signal variation. These results validate the use of AuNP-functionalised detection antibodies for improving the sensitivity of the multiplexed electrochemical immunoassay.

3 Determination of the Limit of Blank and Limit of Detection

The analytical signal decreased with increasing biomarker concentration. Therefore, the limit of blank (LoB) and signal-domain limit of detection (LoD) were calculated using the lower-tail forms of the one-sided 95% criterion. The LoB was calculated as

$$\mathrm{LoB} = \overline{A}_{\mathrm{blank}} - 1.645\sigma_{\mathrm{blank}}, \tag{S1}$$

where $\overline{A}_{\mathrm{blank}}$ and σ_{blank} represent the mean and standard deviation of the blank measurements, respectively. The signal-domain LoD was subsequently calculated as

$$\mathrm{LoD}_{\mathrm{signal}} = \mathrm{LoB} - 1.645\sigma_{\mathrm{low}}, \tag{S2}$$

where σ_{low} is the standard deviation obtained from replicate measurements at the lowest non-zero biomarker concentration. The negative signs were used because increasing biomarker concentration produced a decreasing analytical response. The factor 1.645 corresponds to a one-sided 95% criterion. The blank and lowest-concentration measurements were performed in triplicate ($n = 3$). The resulting signal-domain LoB and LoD thresholds are provided in Table S1.

Table S1: Calculation of the signal-domain limit of blank and limit of detection for PCT, CRP, and SAA in PBS and 50% serum. All values are reported in the signal units used for calibration. Please note that outliers from PCT (both matrix) blank measurements were removed for SD calculations.

**Biomarker**	**Matrix**	**Blank mean**	**Blank SD**	**Lowest-concentration SD**	**LoB**	$\mathbf{LoD_{signal}}$
PCT	PBS	5.62333×10^{-5}	8.20000×10^{-6}	8.13921×10^{-6}	4.27443×10^{-5}	2.93553×10^{-5}
PCT	50% serum	2.66629×10^{-6}	7.24792×10^{-8}	1.58477×10^{-7}	2.54706×10^{-6}	2.28637×10^{-6}
CRP	PBS	7.78000×10^{-6}	1.04022×10^{-6}	4.77517×10^{-7}	6.06884×10^{-6}	5.28332×10^{-6}
CRP	50% serum	4.24632×10^{-6}	7.93494×10^{-8}	4.96546×10^{-8}	4.11579×10^{-6}	4.03411×10^{-6}
SAA	PBS	3.98398×10^{-5}	9.38648×10^{-6}	2.92216×10^{-6}	2.43990×10^{-5}	1.95921×10^{-5}
SAA	50% serum	7.87099×10^{-6}	1.31113×10^{-6}	5.86873×10^{-7}	5.71418×10^{-6}	4.74878×10^{-6}

The calibration equations were obtained by regressing the analytical signal, A, against the base-10 logarithm of biomarker concentration:

$$A = m \log_{10}(C) + b, \tag{S3}$$

where m and b are the calibration slope and intercept, respectively. Here, C represents the numerical concentration expressed in $\mathrm{ng\,mL^{-1}}$ for PCT and $\mu\mathrm{g\,mL^{-1}}$ for CRP and SAA.

Because logarithmically transformed concentrations were used, the signal-domain LoD was converted to a concentration using

$$\log_{10}(C_{\text{LoD}}) = \frac{A_{\text{LoD}} - b}{m}, \tag{S4}$$

followed by

$$C_{\text{LoD}} = 10^{(A_{\text{LoD}} - b)/m}. \tag{S5}$$

All calculations were performed using the unrounded experimental values and full-precision regression coefficients. The calibration equations and corresponding concentration-domain LoDs are shown in Table S2.

Table S2: Conversion of the signal-domain LoD thresholds to concentration-domain LoDs using the corresponding log-linear calibration equations.

**Biomarker**	**Matrix**	**Log-linear calibration equation**	**$\log_{10}(C_{\text{LoD}})$**	**Concentration LoD**
PCT	PBS	$A = -7.349 \times 10^{-6} \log_{10}(C) + 1.765 \times 10^{-5}$	−1.593	0.0255 ng mL^{-1}
PCT	50% serum	$A = -1.476 \times 10^{-7} \log_{10}(C) + 2.411 \times 10^{-6}$	0.844	6.99 ng mL^{-1}
CRP	PBS	$A = -1.440 \times 10^{-6} \log_{10}(C) + 5.577 \times 10^{-6}$	0.204	1.60 $\mu\text{g mL}^{-1}$
CRP	50% serum	$A = -3.531 \times 10^{-7} \log_{10}(C) + 3.591 \times 10^{-6}$	−1.255	0.0556 $\mu\text{g mL}^{-1}$
SAA	PBS	$A = -8.376 \times 10^{-6} \log_{10}(C) + 9.762 \times 10^{-6}$	−1.174	0.0670 $\mu\text{g mL}^{-1}$
SAA	50% serum	$A = -2.112 \times 10^{-6} \log_{10}(C) + 3.240 \times 10^{-6}$	−0.714	0.193 $\mu\text{g mL}^{-1}$

The calculated concentration-domain LoDs were 0.0255 and 6.99 ng mL^{-1} for PCT in PBS and 50% serum, respectively; 1.60 and 0.0556 $\mu\text{g mL}^{-1}$ for CRP; and 0.0670 and 0.193 $\mu\text{g mL}^{-1}$ for SAA. We would also like to highlight that the PCT LoD in 50% serum and the CRP LoD in PBS fell within the experimentally investigated non-zero concentration interval. The calculated PCT LoD in PBS and the CRP and SAA LoDs in 50% serum or PBS, as applicable, fell below the lowest non-zero concentration tested. These values are therefore reported as calculated extrapolations from the corresponding log-linear calibration equations and should not be interpreted as experimentally verified detection limits.

4 Dilution effect

A further control experiment was conducted to verify that the observed signal suppression originated primarily from antibody-antigen binding interactions associated with sandwich complex formation rather than from simple matrix dilution effects alone. Figure S4 investigates how serial dilution influences bioassay performance for all biomarkers in the absence of antigen (PBS buffer blank), in full bioassays, and in 50% serum. The comparison illustrates that signal intensity decreases predictably with increasing dilution, validating that the system responds consistently to concentration changes.

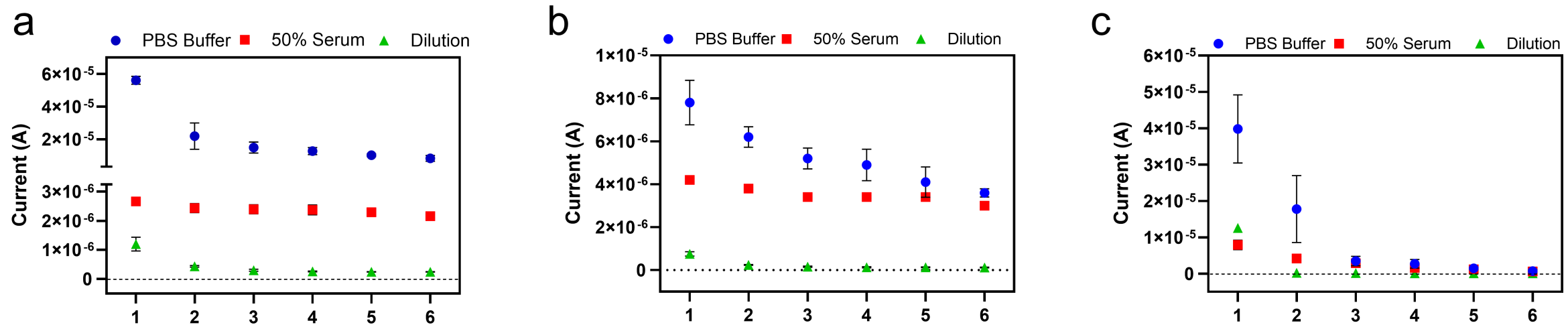

Figure S4: Dilution experiment tested with no antigen present in PBS buffer compared to a full bioassay in PBS buffer and a full bioassay in 50% serum: (a) PCT, (b) CRP, and (c) SAA. The error bars in (a)–(c) correspond to the SD for multiple experimental replicates, $n \geq 3$. X-axis indicates the number of dilutions made

5 Localised surface plasmon resonance measurements and device setup

An image of the localised surface plasmon resonance (LSPR) device configuration has been shown in Figure S5.

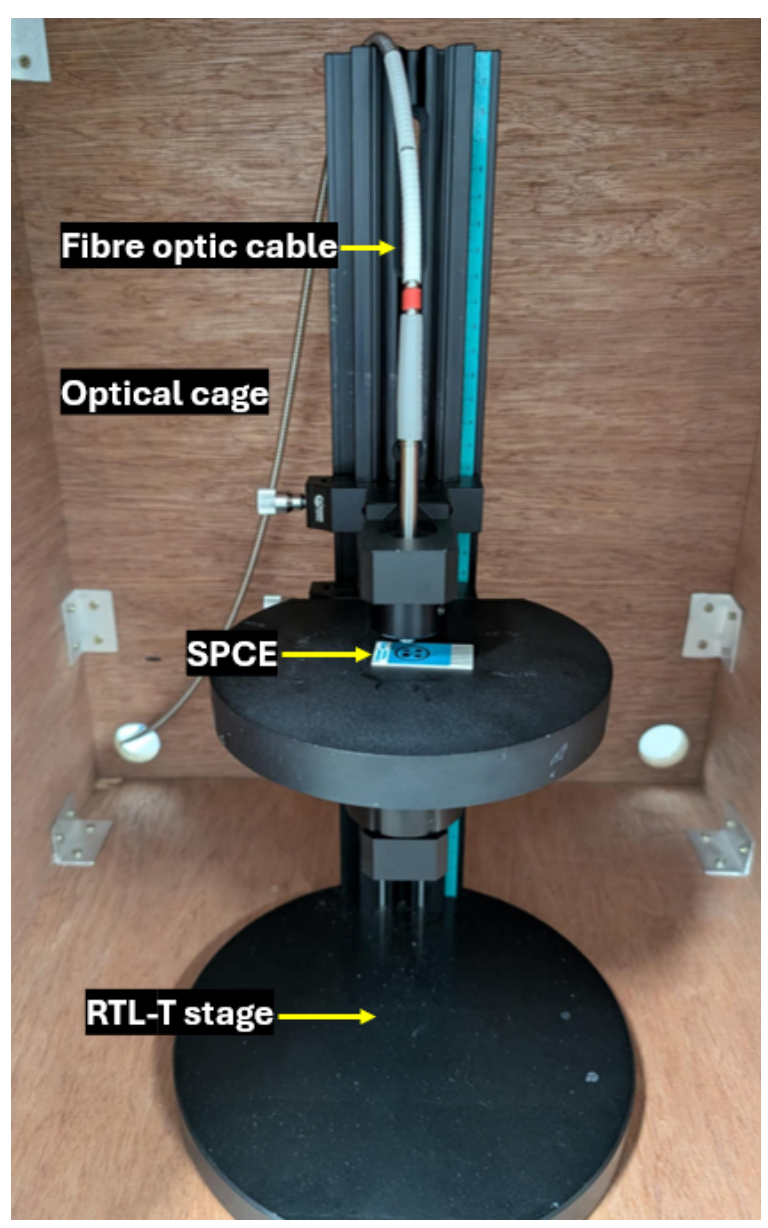

Figure S5: LSPR device setup

Figure S6 shows the LSPR responses for PCT, CRP, and SAA obtained using full bioassays prepared in PBS buffer. Each biomarker produced an increase in absorbance with increase in antigen concentration, indicating successful antigen–antibody binding on the NP-functionalised surface. The magnitude of the increase in absorbance reflects the extent of biomolecular interaction occurring at the plasmonic interface, providing an optical validation of the binding events observed electrochemically. These measurements serve as an indepen-

dent confirmation of bioassay functionality and demonstrate that both electrochemical and optical readouts detect the same antigen-specific interactions.

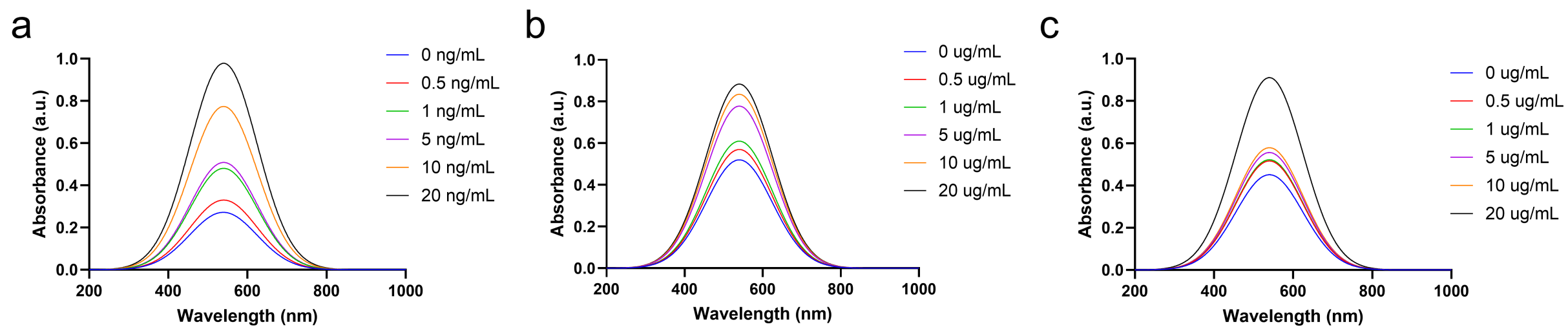

Figure S6: LSPR measurements: a) PCT, b) CRP, and c) SAA. A full bioassay in PBS buffer was used for this experiment.

6 Analytical comparison of the present multiplexed electrochemical–LSPR immunoassay

Table S3: Analytical comparison of the present multiplexed electrochemical–LSPR immunoassay with selected reported multiplex biosensing platforms.

Reference	Biomarkers	Platform	Matrix	Analytical performance
**Present work**	PCT, CRP, SAA	4-WE SPCE differential-pulse voltammetry with complementary LSPR confirmation	PBS; 50% human serum	**Non-zero calibration range:** PCT, 0.5–20 ng mL^{-1}; CRP and SAA, 0.5–20 μg mL^{-1}. Blank samples were analysed separately.<br>**Calculated LoD in PBS:** PCT, 0.0255 ng mL^{-1}[a]; CRP, 1.60 μg mL^{-1}; SAA, 0.0670 μg mL^{-1}[a].<br>**Calculated LoD in 50% serum:** PCT, 6.99 ng mL^{-1}; CRP, 0.0556 μg mL^{-1}[a]; SAA, 0.193 μg mL^{-1}[a].
Bradley *et al.* [1]	PCT, CRP, SAA	AuNP microarray lateral-flow device with optical readout	PBS	**Tested range:** PCT, 0–2 ng mL^{-1}; CRP, 0–2 μg mL^{-1}; SAA, 0–10 μg mL^{-1}.<br>**LoD:** PCT, 0.24 ng mL^{-1}; CRP, 3.25 μg mL^{-1}; SAA, 1.79 μg mL^{-1}.
Lu *et al.* [2]	CRP, SAA	Portable dual-channel electrochemical immunomagnetic-bead biosensor	Plasma	**Linear range:** CRP, 3.125–1250 ng mL^{-1}; SAA, 31.25–1250 ng mL^{-1}.<br>**LoD:** CRP, 0.91 ng mL^{-1}; SAA, 12 ng mL^{-1}.

[a]The calculated concentration-domain LoD lies below the lowest non-zero concentration included in the calibration series. It is therefore an extrapolated analytical estimate obtained from the corresponding log-linear calibration equation rather than an experimentally verified detection limit.

Note: Reported LOD values are not directly comparable across studies because assay architecture, matrix, calibration protocol, and LOD calculation differ. The present study is an analytical proof-of-concept and does not claim clinical diagnostic superiority.

References

[1] Bradley, Z.; McNamee, S. E.; Fishlock, S. J.; Bhalla, N.; Moore, T.; McLaughlin, J. Enhancing Lateral Flow Device Design for Multiplexed Plasmonic Biosensing of Inflammatory Biomarkers. *IEEE Sens. Lett.* **2025**, *9*, 1–4. https://doi.org/10.1109/LSENS.2025.3607559.

[2] Lu, T.-C.; Yang, Y.-J.; Zhong, Y.; Qiu, Q.-Z.; Chen, Z.-H.; Chen, Y.-Z.; Lei, Y.; Liu, A.-L. Rapid Bacterial/Viral Infections Typing Strategy Using a Portable Dual-Channel Electrochemical Biosensor Based on One-Step Assembly of Immunomagnetic Beads. *Anal. Chem.* **2025**, *97*, 5953–5961. https://doi.org/10.1021/acs.analchem.4c03442.
